# Impact of an Early Discharge Hospital-at-Home Program on Length of Stay and Clinical Outcomes in Preterm Infants: A Case–Control Study

**DOI:** 10.3390/children12111504

**Published:** 2025-11-06

**Authors:** María Ángeles García-Ortega, José Miguel García-Piñero, Alberto José Gómez-González, Rosana Medina-López, Marta González-García, Antonio Jesús Montero-García, Isabel María Morales-Gil

**Affiliations:** 1 Hospital Regional Universitario de Málaga, 29009 Málaga, Spain; mariaa.garcia.ortega.sspa@juntadeandalucia.es (M.Á.G.-O.); jgp@uma.es (J.M.G.-P.); 2 Department of Nursing, Faculty of Health Sciences, University of Malaga, 29071 Málaga, Spain; mgonzalezgarcia@uma.es (M.G.-G.); monterogarciaantonio@uma.es (A.J.M.-G.); im_morales@uma.es (I.M.M.-G.); 3 Instituto de Investigación Biomédica de Málaga y Plataforma en Nanomedicina (IBIMA Plataforma Bionand), 29590 Málaga, Spain; 4 Hospital Universitario Costa del Sol, 29603 Marbella, Spain; rosana.medina.sspa@juntadeandalucia.es; 5 Health Care Emergency Center 061, 29590 Málaga, Spain

**Keywords:** preterm infants, hospital-at-home, early discharge, breastfeeding, kangaroo mother care, neonatal nursing, length of stay

## Abstract

**Highlights:**

**What: are the main findings?**

- Hospital-at-home (HaH) after early discharge was associated with a −5.5-day adjusted reduction in hospital stay (β −5.53; 95% CI −10.96 to −0.11; *p* = 0.046).
- Longer exclusive breastfeeding duration was observed in the HaH group (142 vs. 81 days; *p* = 0.024), with no increase in complications or acute service use.

**What are the implications of the main findings?**

- HaH programs were associated with shorter hospital stays without compromising safety, promoting family-centered care.
- The role of neonatal nursing is essential for ensuring a safe transition and supporting breastfeeding/kangaroo mother care at home.

**Abstract:**

**Background/Objectives**: Prolonged hospitalization of clinically stable preterm infants may lead to nosocomial infections, interfere with breastfeeding, and hinder parent-infant bonding. We evaluated the impact of an early discharge program with hospital-at-home (HaH) on hospital stay and clinical outcomes among preterm infants. **Methods**: A retrospective case–control study was conducted in a tertiary neonatal unit (Spain). Fifty infants managed with HaH (2016–2020) were compared with ninety-six controls receiving conventional in-hospital care. Baseline characteristics, growth, and clinical events up to 12 months were collected. Analyses included bivariate comparisons and multiple linear regression for length of stay, adjusted for gestational age, birth weight, sex, and parental factors. **Results**: Baseline characteristics were comparable between groups. Discharge weight was lower in HaH infants (1865 vs. 2130 g; *p* < 0.001), but no differences were observed at 6 or 12 months. Length of stay was shorter in HaH infants (26.3 vs. 33.8 days; *p* = 0.081), and the multivariable model showed an independent 5.5-day reduction (β −5.53; 95% CI −10.96 to −0.11; *p* = 0.046). Exclusive breastfeeding was more frequent (74% vs. 59%; *p* = 0.08) and significantly longer in HaH infants (141.9 vs. 81.1 days; *p* = 0.024). No increases were found in complications at discharge, emergency visits (28% vs. 32%; *p* = 0.7), or readmissions (18% vs. 31%; *p* = 0.2). **Conclusions**: Among clinically stable preterm infants, early discharge with HaH was associated with a shorter hospital stay and longer exclusive breastfeeding duration, without evidence of increased morbidity or healthcare use; however, causal inference cannot be established due to the observational design. These findings support the implementation of nurse-led HaH programs as a safe, family-centered strategy for neonatal care.

## 1. Introduction

Prematurity represents a significant public health problem due to its high morbidity, mortality, and the associated economic and social burden. According to the World Health Organization (WHO), newborns born before completing 37 weeks of gestation are considered premature. This condition is the leading cause of neonatal morbidity and the second leading cause of death in children under five years of age [1], affecting approximately one in ten newborns worldwide.

The determinants of preterm birth are diverse and complex, encompassing biological, environmental, social, economic, and political factors. This multifactorial nature requires coordinated responses from health systems and governments through public policies aimed at the prevention and comprehensive management of prematurity [2,3].

The physiological immaturity of preterm infants exposes them to severe complications requiring specialized care [4]. However, prolonged hospitalization—particularly during clinically stable phases—can lead to adverse effects such as nosocomial infections, disruption of breastfeeding, diagnostic overload, and interference with family bonding [5,6]. Moreover, extended stays in neonatal units often represent a crisis experience for families, hindering the establishment of parental roles and emotional attachment [7].

In this context, several high-income countries have implemented early discharge programs supported by Hospital-at-Home (HaH) care [8,9,10], aimed at continuing neonatal care within the family environment once clinical stability has been achieved. Evidence from countries such as Sweden, France, the United States, and Spain suggests that this care model reduces hospital stay without compromising neonatal outcomes, while promoting the humanization of care and empowering families [8,11,12].

Home-based care is a cost-effective and family-centered strategy in which nursing professionals play a key role in ensuring care continuity and discharge safety [13,14,15]. Despite the positive outcomes reported in international literature, there is a need for context-specific evidence, particularly in healthcare settings where this model is not yet fully established.

Therefore, the present study aims to evaluate the impact of an early discharge program with HaH care on hospital length of stay and the clinical outcomes of preterm infants. The specific objectives are: (1) to compare parental sociodemographic and neonatal baseline characteristics between the HaH and conventional discharge groups; (2) to analyzed the impact of the program on hospital stay, weight gain, and exclusive breastfeeding; (3) to assess the use of kangaroo mother care (KMC) in both groups; and (4) to identify clinical complications, emergency visits, rehospitalizations, and healthcare utilization during the first year of life, in order to evaluate the safety of this care strategy.

## 2. Materials and Methods

### 2.1. Study Design

A case–control study was conducted to evaluate the impact of a HaH program on hospital length of stay and clinical outcomes in preterm infants. Two groups were compared: an exposed group that received early discharge with HaH care, and a control group managed with conventional in-hospital care. A 2:1 matching by sex, gestational age (±5 days), and birth weight (±100 g) was performed between infants. This ratio was selected to increase statistical power while maintaining comparable baseline characteristics across groups.

All eligible preterm infants who participated in the HaH program during the study period were included. Controls were selected consecutively from the same unit and time frame, matched according to the specified criteria.

Both groups were drawn from the same neonatal unit and time period (January 2016–December 2020), ensuring temporal overlap in case and control selection. Sociodemographic and family-related variables, including parental educational level and parity (number of previous children), were collected to assess potential socioeconomic or behavioral differences between groups. Although matching minimized clinical variability, residual socio-behavioral differences cannot be fully excluded.

This study adheres to the Strengthening the Reporting of Observational Studies in Epidemiology (STROBE) statement for case–control studies, and the corresponding checklist is provided as Supplementary Table S1.

### 2.2. Study Setting and Population

The study was conducted in the Neonatology Unit of the Hospital Materno-Infantil of Málaga, Spain. The exposed group included 50 preterm infants who participated in the HaH program between January 2016 and December 2020. The non-exposed (control) group consisted of 96 preterm infants admitted to the same unit who met clinical criteria for discharge but did not participate in the HaH program.

### 2.3. Inclusion and Exclusion Criteria

The study included preterm infants (gestational age < 37 weeks), provided they met the same clinical criteria for hospital discharge in both groups to ensure baseline comparability. These criteria included clinical stability with normal vital signs and spontaneous breathing in room air; absence of apnea or bradycardia episodes for at least five days, with no ongoing pharmacological treatment for apnea of prematurity; the ability to maintain body temperature in an open crib; and effective oral feeding through suction. In addition, families had to show adequate caregiving capacity after in-hospital education and reside within 25 kilometers of the hospital in a home with appropriate physical conditions and telephone access. Infants were excluded if they were presented with major congenital anomalies, known genetic syndromes, or if the family declined participation in follow-up.

### 2.4. Exposure

The HaH program is an early discharge model aimed at delivering hospital-level care in the infant’s home environment. It was managed by a multidisciplinary team composed of two pediatricians and two pediatric nurses specialized in neonatal care. The HaH program consisted of structured health education sessions for parents before discharge, scheduled home visits adjusted to the infant’s clinical needs, and continuous telephone follow-up to provide support and monitor progress. Parental education focused on understanding the characteristics of prematurity, including the distinction between chronological and corrected age, as well as breastfeeding support, KMC, and preparation for the transition home. Families were also provided with written guidance addressing feeding, sleep, weight monitoring, temperature control, prevention of sudden infant death syndrome (SIDS), infection prevention, and the identification of clinical warning signs [16].

The HaH team consisted of two pediatricians and two pediatric nurses specialized in neonatal care, each with more than ten years of experience in neonatal intensive care and formal training in breastfeeding counseling and KMC. The HaH program included scheduled home visits (typically two to three per week during the first two weeks, and subsequently according to the infant’s condition), 24 h telephone access for clinical queries or emergencies, and coordination with primary care services. Continuous follow-up allowed individualized support, reinforcement of parental education, and early detection of complications.

### 2.5. Data Collection

Data were obtained through a retrospective review of electronic medical records and structured telephone interviews with parents or caregivers who provided informed consent. The data collection was carried out between January 2021 and June 2021, corresponding to infants discharged between 1 January 2016, and 31 December 2020. Variables collected included demographic and perinatal characteristics, hospital stay duration, post-discharge complications, and readmissions.

### 2.6. Statistical Analysis

Quantitative variables were expressed as means and standard deviations (SD); qualitative variables were described as frequencies and percentages. Comparisons between groups were made using Chi-square tests, Student’s *t*-tests, or ANOVA, depending on variable type and distribution. When assumptions of normality were violated, non-parametric tests were used. Pearson or Spearman correlations were applied as appropriate. A *p*-value < 0.05 was considered statistically significant, with 95% confidence intervals (CI) reported where applicable.

To further evaluate the independent effect of the HaH program on hospital stay, a multiple linear regression model was performed, adjusting for potential confounders including gestational age, birth weight, sex, mother’s and father’s age, and parental educational level. Model fit was assessed using the coefficient of determination (R^2^) and statistical significance of predictors was reported with β coefficients, 95% CI, and *p*-values.

All analyses were conducted using Jamovi 2.6, an open-source statistical software [17]. No missing data were present in the analyzed variables.

### 2.7. Ethical Considerations

The study was approved by the Malaga Provincial Research Ethics Committee (approval code 0208-N-19, approval date 28 January 2021) (Spain). Patient consent was waived due to the retrospective nature of the study and the use of anonymized patient records. The research was conducted in accordance with the ethical principles of the Declaration of Helsinki (1975, revised 2013) for medical research involving human subjects and complied with the requirements of the Spanish Organic Law 3/2018 on the Protection of Personal Data and Guarantee of Digital Rights. Confidentiality and anonymity of all participants were rigorously maintained throughout the study.

### 2.8. Data Availability

The datasets generated and analyzed during the current study are available from the corresponding author upon reasonable request. No public database was used. There are no restrictions on material or data availability.

### 2.9. Use of Generative AI

ChatGPT-4.0 (OpenAI) was used for language editing and grammar correction to enhance clarity and accuracy in the English version of the manuscript. No AI tools were employed for data analysis, content generation, or the interpretation of the research findings.

## 3. Results

A total of 146 infants were included, with 96 in the control group and 50 in the HaH group. There was no missing data. No significant differences were observed between groups in maternal age (33.45 ± 5.8 years), paternal age (36.36 ± 6.26 years), parental educational level, or parity. Parental assessment of the information provided by neonatology and nursing staff was overall positive in both groups (Table 1).

Mean gestational age was 32.2 ± 2.7 weeks and mean birth weight was 1488 g, with no significant differences between groups (Table 2). Sex distribution was balanced. At hospital discharge, infants in the HaH group had a significantly lower mean weight (1864 g) compared to controls (2129 g; *p* < 0.001). However, this difference disappeared at 6 and 12 months of follow-up. Hospital stay was shorter in the HaH group (26.3 vs. 33.8 days), although the difference did not reach statistical significance in the bivariate analysis (*p* = 0.081). Exclusive breastfeeding was more frequent in the HaH group (74% vs. 59%), although this difference did not reach statistical significance (*p* = 0.08). This may partly reflect greater parental motivation or preference for participation in the HaH program rather than a direct effect of the intervention. The duration of exclusive breastfeeding, however, was significantly longer in the HaH group (141.9 vs. 81.1 days; *p* = 0.024). KMC was widely applied in both groups, with a mean duration of 24 days.

Regarding safety outcomes, at hospital discharge 48% of infants in the HaH group presented some health problems compared with 57% in the control group (*p* = 0.4) (Table 3). The most frequent complications were respiratory (24% in HaH vs. 31% in control), followed by digestive (10% vs. 15%) and neurological (6% vs. 8%), with no statistically significant differences. These findings suggest that early discharge with HaH does not increase the immediate risk of clinical complications. During the first year of life, emergency department visits were similar between groups (28% in HaH vs. 32% in control; *p* = 0.7), indicating that the strategy did not generate additional acute healthcare demand. Subsequent hospitalizations were less frequent in the HaH group (18% vs. 31% in control; *p* = 0.2), a non-significant difference but one that suggests a favorable trend supporting the safety of the program. Primary care utilization was comparable between groups (18% in HaH vs. 30% in control; *p* = 0.9), reflecting that community follow-up after early discharge remained aligned with conventional discharge care.

The multiple linear regression model explained 64.8% of the variability in hospital stay (R^2^ = 0.648) (Table 4). In this analysis, HaH care was independently associated with a significant reduction of 5.5 days compared to the control group (β = −5.53; 95% CI: −10.96 to −0.11; *p* = 0.046). Gestational age and birth weight also emerged as key predictors: each additional week of gestation reduced hospital stay by 5.1 days (*p* < 0.001), and each additional 100 g of birth weight by 1.5 days (*p* = 0.002). These results are clinically consistent with the natural course of prematurity and reinforce the robustness of the model. Conversely, sex, parental age, and educational level showed no significant association with hospital stay, ruling out confounding by sociodemographic factors. Overall, this multivariate analysis confirms that participation in the HaH program is an independent factor associated with shorter hospital stay, without compromising neonatal safety.

## 4. Discussion

This study aimed to evaluate the impact of an early discharge program with HaH care on hospital stay and clinical outcomes in preterm infants. The analysis also sought to compare baseline sociodemographic and neonatal characteristics between groups, examine growth and breastfeeding patterns, and assess safety in terms of complications, healthcare utilization, and readmissions during the first year of life.

The results demonstrate that these objectives were met, showing that HaH care is an effective and safe alternative to conventional hospitalization, promoting family-centered neonatal care.

### 4.1. Hospital Stay and Associated Factors

The multiple linear regression model explained 64.8% of the variance in hospital stay (R^2^ = 0.648; model *p* < 0.001), confirming the robustness of the analysis. Participation in the HaH program independently predicted a 5.5-day reduction in length of stay (95% CI: −10.96 to −0.11; *p* = 0.046). Gestational age and birth weight were also significant predictors, consistent with previous studies reporting similar associations. These findings highlight the effectiveness of HaH programs in facilitating safe early discharge, without compromising neonatal stability or continuity of care [18,19].

### 4.2. Growth and Weight Evolution

Although infants in the HaH group were discharged at a lower mean weight (1864 g vs. 2129 g; *p* < 0.001), no significant differences were found at 6 or 12 months, confirming adequate catch-up growth. This aligns with evidence showing that preterm infants achieve compensatory growth when adequate nutritional and lactation support are provided [20]. The broad use of KMC (mean duration 24 days) likely contributed to this favorable progression by improving thermoregulation and metabolic adaptation [21,22].

### 4.3. Breastfeeding and Parental Bonding

Exclusive breastfeeding prevalence and duration were higher in the HaH group (74% vs. 59%; *p* = 0.08 and 142 vs. 81 days; *p* = 0.024, respectively). Although the higher prevalence was not statistically significant, this finding might reflect self-selection of families more motivated or prepared for early discharge rather than a causal program effect. The longer duration of exclusive breastfeeding observed among HaH infants is consistent with previous studies suggesting that home-based transitional care can help sustain breastfeeding by fostering parental autonomy and minimizing institutional disruption [23,24]. The home environment also facilitates continuous skin-to-skin care and reduces maternal stress, enhancing both nutritional and emotional outcomes for families.

### 4.4. Clinical Safety and Healthcare Utilization

No significant differences were observed in complications at discharge (48% vs. 57%; *p* = 0.4), and the distribution of respiratory, digestive, and neurological conditions was comparable between groups. Emergency visits were similar (28% vs. 32%; *p* = 0.7), and rehospitalizations were less frequent in the HaH group (18% vs. 31%; *p* = 0.2), suggesting a clinically relevant trend toward fewer readmissions. Primary care utilization was equivalent, indicating adequate coordination between hospital and community services. These results confirm the clinical safety of the HaH model, with no evidence of increased morbidity or healthcare burden [25].

### 4.5. Role of the Neonatal Nurse

A cornerstone of the HaH model’s success is the advanced role of neonatal nurses as coordinators of home-based care. Their involvement ensured safe monitoring of growth, feeding, and thermoregulation, as reflected in the comparable outcomes between groups. Moreover, their leadership in family education and KMC continuity strengthened parental confidence and competence. These findings reinforce the value of specialized neonatal nursing within transitional care frameworks, promoting developmental, psychosocial, and family-centered outcomes [14,15,26].

### 4.6. Overall Interpretation

In summary, all study objectives were addressed: baseline comparability between groups ensured internal validity; HaH participation independently reduced hospital stay; growth and breastfeeding outcomes remained favorable; and no increase in complications or healthcare use was detected. Collectively, these findings demonstrate that HaH programs offer a safe, effective, and family-centered approach to neonatal care, aligned with the goals of humanized medicine and healthcare efficiency.

### 4.7. Limitations

This study has several limitations. The relatively small sample size may limit the statistical power and generalizability of the results. The retrospective design relies on the accuracy of medical records and does not allow for causal inference. Additionally, potential selection bias may exist, as families opting for HaH might have stronger social support or motivation. Although a 2:1 matching design improved precision, residual confounding cannot be completely excluded due to the retrospective nature of the study. Future multicenter prospective studies could confirm these findings in larger and more diverse populations. Finally, long-term neurodevelopmental outcomes and family well-being were not systematically assessed and should be explored in future studies.

## 5. Conclusions

This study demonstrates that early hospital discharge of preterm infants, supported by a structured HaH program, is a safe and effective alternative to conventional care. Participation in HaH was independently associated with a significant reduction in hospital stay and longer exclusive breastfeeding duration, without increasing morbidity or healthcare utilization.

The pivotal role of neonatal nurses was central to program success, ensuring clinical safety, promoting breastfeeding, and facilitating family empowerment. These findings support the progressive implementation of nurse-led transitional care programs integrating the home as a safe therapeutic environment that enhances neonatal outcomes and family engagement.

Future research should evaluate long-term neurodevelopmental and psychosocial outcomes and examine the scalability and sustainability of HaH models across diverse healthcare systems.

## Figures and Tables

**Table 1:** Descriptive analysis of parents and bivariate analysis by group.

Characteristic		Overall *n* = 146	Control *n* = 96	HaH *n* = 50	*p*-Value *
Mother’s age	Years	33.45 (5.80)	32.98 (5.33)	34.34 (6.56)	0.18 ^‡^
Father’s age	Years	36.36 (6.26)	35.99 (5.83)	37.08 (7.04)	0.33 ^‡^
Mother’s level of education	Primary education	12 (8.3%)	9 (9.5%)	3 (6%)	0.4 ^†^
	Secondary education	33 (23%)	22 (23%)	11 (22%)	
	Vocational training	46 (32%)	33 (35%)	13 (26%)	
	University education	55 (36.7%)	32 (32.5%)	23 (46%)	
Father’s level of education	Primary education	18 (12.3%)	13 (13.5%)	5 (10%)	0.6 ^†^
	Secondary education	41 (28.1%)	26 (27.1%)	15 (30%)	
	Vocational training	42 (28.8%)	26 (27.1%)	16 (32%)	
	University education	45 (30.8%)	31 (32.3%)	14 (28%)	
Nurse information	Very poor	4 (2.74%)	3 (3.13%)	1 (2%)	0.21 ^†^
	Poor	5 (3.42%)	4 (4.17%)	1 (2%)	
	Average	9 (6.16%)	8 (8.3%)	1 (2%)	
	Good	19 (13.01%)	9 (9.38%)	10 (20%)	
	Very good	109 (74.67%)	72 (75.02%)	37 (74%)	
Neonatologist information	Very poor	2 (1.37%)	2 (2.08%)	0 (0%)	0.63 ^†^
	Poor	2 (1.37%)	2 (2.08%)	0 (0%)	
	Average	7 (4.79%)	5 (5.21%)	2 (4%)	
	Good	17 (11.64%)	10 (10.42%)	7 (14%)	
	Very good	118 (80.83%)	77 (80.21%)	41 (82%)	
Previous children	0	85 (58.22%)	58 (60.42%)	27 (54%)	0.2 ^†^
	1	36 (24.66%)	24 (25%)	12 (24%)	
	2	20 (13.7%)	10 (10.42%)	10 (20%)	
	3	4 (2.74%)	4 (4.17%)	0 (0%)	
	4	1 (0.68%)	0 (0%)	1 (2%)	

Note: * *p*-value < 0.05 = statistical significance; ^†^ Chi-squared test; ^‡^ Student *t*-test.

**Table 2:** Descriptive analysis of newborns and bivariate analysis by groups.

Characteristic		Overall *n* = 146	Control *n* = 96	HaH *n* = 50	*p*-Value *
Gestational age	Weeks	32.2 (2.71)	31.9 (2.8)	32.7 (2.47)	0.09 ^‡^
Sex	Male	65 (44.5%)	43 (44.79%)	22 (44%)	0.93 ^†^
	Female	81 (55.5%)	53 (55.21%)	28 (56%)	
Birth weight	g	1488.4 (412.15)	1504.1 (427.95)	1458.1 (382.33)	0.52 ^‡^
Weight at hospital discharge		2036.3 (342.32)	2129.7 (334.04)	1864.6 (288.98)	<0.001 ^‡^
Weight at 6 months		5570 (1320)	5630 (1360)	5470 (1270)	0.57 ^‡^
Weight at 12 months		8010 (1540)	8050 (1710)	7940 (1190)	0.75 ^‡^
Hospital stay	Days	31.2 (24.65)	33.8 (26.33)	26.3 (20.38)	0.081 ^‡^
Exclusive breastfeeding	Yes	94 (64.4%)	57 (59.38%)	37 (74%)	0.08 ^†^
	No	52 (35.6%)	39 (40.62%)	13 (26%)	
Exclusive breastfeeding time	Days	101.9 (155.2)	81.1 (131.72)	141.9 (187.49)	0.024 ^‡^
KMC	Yes	124 (84.9%)	80 (%)	44 (88%)	0.45 ^†^
	No	22 (15.1%)	16 (%)	6 (12%)	
KMC time	Days	24.0 (21.38)	24.1 (20.46)	23.9 (23.25)	0.95 ^‡^

Note: grams (g); kangaroo mother care (KMC); * *p*-value < 0.05 = statistical significance; ^†^ Chi-squared test; ^‡^ Student *t*-test.

**Table 3:** Descriptive analysis of health problems and healthcare utilization after hospital discharge and bivariate analysis by group.

Characteristic		Control *n* = 96	HaH *n* = 50	*p*-Value *
Health problems after hospital discharge	Yes	55 (57%)	24 (48%)	0.4 ^†^
	No	41 (43%)	26 (52%)	
Health issues	Respiratory	30 (31.3%)	12 (24%)	0.7 ^†^
	Digestive	6 (6.2%)	4 (8%)	
	Neurological	7 (7.2%)	4 (8%)	
	Hematological	4 (4.1%)	1 (2%)	
	Cardiac	4 (4.1%)	1 (2%)	
	Others	4 (4.1%)	2 (4%)	
Visit to emergency departments	Yes	31 (32%)	14 (28%)	0.7 ^†^
	No	65 (68%)	36 (72%)	
Subsequent hospital admissions	Yes	29 (31%)	9 (18%)	0.2 ^†^
	No	66 (69%)	40 (82%)	
Visit to health centers	Yes	29 (30.2%)	9 (18%)	0.9 ^†^
	No	67 (69.8%)	41 (82%)	

Note: * *p*-value < 0.05 = statistical significance; ^†^ Chi-squared test.

**Table 4:** Linear regression model of hospital stay in days as the dependent variable.

Predictor	β	EE	95% CI		t	*p*-Value *
			Lower	Upper		
Constant	210.12	21.14	168.3	251.94	9.94	<0.001
HaH	−5.53	2.74	−10.96	−0.11	−2.02	0.046
Gestational age	−5.08	0.75	−6.57	−3.59	−6.75	<0.001
Birth weight	−0.02	0.01	−0.02	−0.01	−3.22	0.002
Sex	2.81	2.49	2.12	7.75	1.13	0.26
Mother’s level of education (secondary-primary)	4.17	5.5	−6.71	15.04	0.76	0.45
Mother’s level of education (vocational training-primary)	−2.34	5.52	−13.26	8.58	−0.42	0.67
Mother’s level of education (university-primary)	1.36	5.55	−9.63	12.34	0.25	0.81
Father’s level of education (secondary-primary)	3.12	4.9	−6.57	12.81	0.64	0.53
Father’s level of education (vocational training-primary)	3.04	5.15	−7.15	13.23	0.59	0.56
Father’s level of education (university-primary)	1.07	5.16	−9.14	11.29	0.21	0.84
Mother’s age	0.26	0.31	−0.35	0.88	0.84	0.4
Father’s age	−0.13	0.29	−0.7	0.44	−0.45	0.65

Note: R^2^ = 0.648; dependent variable = Hospital stay (days). HaH = hospital-at-home; β = unstandardized coefficient; EE = standard error; 95% CI = 95% confidence interval; t = *t*-value of the model; * *p*-value < 0.05 = statistical significance.

## Data Availability

The datasets generated and analyzed during the current study are available from the corresponding author upon reasonable request. No public database was used. There are no restrictions on material or data availability.

## Supplementary Materials

The following supporting information can be downloaded at: https://www.mdpi.com/article/10.3390/children12111504/s1, Supplementary Table S1: STROBE Statement—Checklist of items that should be included in reports of ***case-control studies***.

## Abbreviations

The following abbreviations are used in this manuscript:

CI	Confidence Interval
HaH	Hospital-at-Home
KMC	Kangaroo Mother Care
SD	Standard Deviation
SIDS	Sudden Infant Death Syndrome
WHO	World Health Organization
